# Are implicit motives revealed in mere words? Testing the marker-word hypothesis with computer-based text analysis

**DOI:** 10.3389/fpsyg.2013.00748

**Published:** 2013-10-16

**Authors:** Oliver C. Schultheiss

**Affiliations:** Department of Psychology, Friedrich-Alexander UniversityErlangen, Germany

**Keywords:** linguistic analysis, implicit motives, picture story exercise, thematic apperception, activity inhibition, personality, assessment

## Abstract

Traditionally, implicit motives (i.e., non-conscious preferences for specific classes of incentives) are assessed through semantic coding of imaginative stories. The present research tested the marker-word hypothesis, which states that implicit motives are reflected in the frequencies of specific words. Using Linguistic Inquiry and Word Count (LIWC; Pennebaker et al., 2001), Study 1 identified word categories that converged with a content-coding measure of the implicit motives for power, achievement, and affiliation in picture stories collected in German and US student samples, showed discriminant validity with self-reported motives, and predicted well-validated criteria of implicit motives (gender difference for the affiliation motive; in interaction with personal-goal progress: emotional well-being). Study 2 demonstrated LIWC-based motive scores' causal validity by documenting their sensitivity to motive arousal.

## Introduction

Implicit motives, that is, non-consciously operating dispositions to experience certain classes of incentives as affectively charged, are assessed through the content coding of written text (Schultheiss and Pang, 2007; Schultheiss, 2008). The traditional measure of implicit motives, the Picture Story Exercise (PSE; McClelland et al., 1989), requires research participants to write 5-min imaginative stories about each of a series of 4–8 pictures showing people in various social situations. The stories are subsequently analyzed using coding systems derived from experimental-arousal studies of motivational needs (Smith, 1992). After careful learning of the coding rules and applying them to training materials, coders are required to pass a high threshold of coding reliability, usually >85% agreement with expert-scored calibration stories, before they can apply their skills to new stories harvested in empirical studies (Schultheiss and Pang, 2007). The coding of a set of stories from a given participant requires between 20 and 30 min. To document interrater reliability in research reports, usually two coders analyze all stories independently.

Thus, although the assessment of implicit motives is objective, as reflected in high agreements among experienced coders and between coders and expert-coded calibration materials, it is also a labor-intensive process that requires thorough training of coders and about 2 (coders) × 25 min for each participant's set of stories. For PSEs from a sample of 100 participants, this means that more than 80 h have to be invested in the content-coding process itself, not counting the time needed for coder training. This is a considerable investment of resources into the assessment of implicit motives, and high interrater reliability is not necessarily guaranteed, especially for coders who are new to the job. These hurdles may appear prohibitive to researchers who want to use implicit motive measures in their research.

In the present paper, I therefore explore to what extent content-coding of PSEs can be approximated by computer-based coding of *marker words, that is, words whose occurrence is indicative of the presence or absence of an implicit motivational need (marker-word hypothesis)*. More specifically, I will examine whether motive scores derived from content coding converge replicably in US and German samples with linear combinations of word categories implemented in the LIWC 2001 software (Pennebaker et al., 2001). I will also examine whether, compared to content-coding scores, LIWC-based scores have comparable discriminant and predictive validity, and whether they are similarly sensitive to experimental arousal.

### The content-coding measurement approach and the marker-word hypothesis

Implicit motive measures have been derived by experimentally arousing a motivational state in one group of participants, leaving it unaroused in a control group and then having both groups write imaginative stories about the same picture cues (see Atkinson, 1958; Smith, 1992). Narrative themes (“imagery”) that occurred more frequently in the arousal than in the control group were then distilled into coding systems. Their coding rules usually deal with the meaning of text at a semantic level; that is, whether imagery is coded or not usually depends on the meaning of an entire sentence or partial sentence, not on the presence or absence of individual words.

Several separate coding systems were derived in this manner to assess the needs for achievement (*n* Achievement; a capacity for enjoying the mastery of challenging tasks; e.g., McClelland et al., 1953), power (*n* Power; a capacity for enjoying one's impact on others; e.g., Winter, 1973), or affiliation (*n* Affiliation; a capacity for enjoying close, harmonious relationships; e.g., Atkinson et al., 1958). The coding system used most frequently in contemporary research is the running-text system by Winter (1991), which, by integrating several earlier coding systems, allows to code all three motives in one run and which can be used with the PSE and other types of texts, such as political speeches or historical documents. Winter (1991) has validated the running-text system by ensuring that the way each motive is coded sensitively differentiates between arousal- and control-group PSEs of the original arousal studies. He thus documented the integrated coding system's causal validity (see Borsboom et al., 2004). He also demonstrated high convergent validity between motive scores coded with the running-text system and scores obtained with the original coding systems.

Because the research reported here was conducted with Winter's (1994) running-text system, I will briefly describe the rules according to which motive imagery is coded with it. In general, story characters' past, present, or future wishes, feeling states, intentions, or actions can be scored as motive imagery. *n* Power is coded whenever a story character (1) acts in a strong, forceful manner, (2) controls or manipulates others, (3) tries to persuade or convince others, (4) provides unsolicited help or advice, (5) wants to impress others, has a concern with fame, prestige, or (6) elicits strong, non-reciprocal emotions in others. *n* Achievement is coded whenever (1) someone's performance is qualified with positive adjectives (e.g., “excellent,” “good”), (2) someone's goals or performances are evaluated positively, (3) winning or competing with others is mentioned, (4) someone shows a negative affective response to failure, or (5) someone makes a unique accomplishment. *n* Affiliation is coded when someone (1) experiences positive affect in the context of a relationship, (2) experiences negative affect about the disruption of a relationship and wants to mend it, (3) engages in companionate activity with others, and (4) provides nurturant support to others.

This brief review of Winter's (1994) coding system shows that most of the themes that can be coded are based on a complex semantic analysis of the text material. The coding of single words, like in the first category of *n* Achievement, is a rare exception from the rule and already reflects a considerable simplification of the coding rules from the original coding systems, which focused exclusively on sentence- or even story-level meaning, to the running-text system. The following example provides an illustration of coding for motivational imagery with Winter's (1994) system (coded themes are underlined; abbreviated motive labels and subcategories are provided in the margin):

**Table:** 

Jim Callahan is famous for saving the crew of the Serendipity from certain death when the vessel collided with a reef out in the open sea. He is also a	*n* Pow (4/5)


highly accomplished captain. Here we can see a passenger who tries to persuade Callahan to admit him for free. The passenger is missing his wife and kids in Europe and is longing to be reunited with them.	*n* Ach (1)
	*n* Pow (3)
	*n* Aff (2)


Can motive assessment be simplified by identifying and counting marker words, that is, words that capture roughly the same meaning that a human coder would see when coding motivational imagery? For instance, in the text above, “accomplished” might be such a marker word for *n* Achievement, because it would be scored by a trained coder and a computer could also easily identify it. In the first sentence, perhaps only the word “famous” would qualify as a marker word for the fifth coding rule of *n* Power (concern with fame and prestige), because at the semantic level most of the other words (e.g., “save,” “crew,” “serendipity”) are not closely related to the need for having impact. Only their complex and highly unique combination in the first sentence would also make them a candidate for coding *n* Power according to the fourth rule (unsolicited help). “Missing” in the last sentence looks like a marker word for *n* Affiliation (second coding rule) at first blush. But the verb *miss* can have several, very different meanings, as in misplacing something or as the opposite of hitting something, and may therefore be too ambiguous as a marker word for *n* Affiliation. Perhaps “wife” would be suitable, because it suggests a certain degree of closeness, whereas “kids” might again be too ambiguous a word, because in other contexts it might simply refer to young or irresponsible people.

This brief analysis highlights the possibilities and ambiguities inherent in finding suitable marker words that could approximate content coding of text. It also suggests that in some instances there *are* words, like “accomplished,” that appear to be unambiguously associated with coded imagery. There have already been several systematic forays into substituting content coding with the word-count approach suggested here.

### Previous studies

Early attempts at measuring motives through marker words include Zatzkis's (1949; cited in McClelland et al., 1953, pp. 249–254) work on the relationship of *n* Achievement with the use of abstract nouns and the omission of negations and hedged statements, Wanner's (1972) study of the relationship between *n* Power and words related to hunting, vigorous activity, possessions, and war in folk tales, and (1965; reported in Smith, 1968) doctoral work on the convergent and predictive validity of content-coded *n* Achievement and achievement-related marker words in the PSE.

Smith (1968) later built on Litwin's work by developing dictionaries for the targeted assessment of *n* Achievement, *n* Power, and *n* Affiliation. Each dictionary consisted of about 20 categories, with each containing 5–150 words. Thus, for instance, the *n* Power dictionary featured the categories *Influence verbs and verb phrases* and *Power activity*, with the former covering words such as “influence,” “win over,” “use pressure” and the latter covering words such as “power play,” “controversy,” and “struggle.” The categories were designed to represent each coding category of the original coding systems. Using the General Inquirer system by Stone et al. (1966), Smith then applied the dictionaries to stories contained in Atkinson (1958) as well as to new stories written to novel pictures. Agreement, as assessed by Pearson's *r*, between experts' content-codings of the stories with dictionary-derived scores ranged from 0.73 to 0.87 for *n* Achievement, from 0.61 to 0.84 for *n* Affiliation, and from 0.41 to 0.73 for *n* Power. Smith concluded that dictionary-based coding worked rather well for *n* Achievement and *n* Affiliation, but not for *n* Power.

Seidenstücker and Seidenstücker (1974) aimed at creating a custom-tailored dictionary for the assessment of *n* Achievement, with the goal of maximizing convergent validity with Heckhausen's (1963) coding system. However, they used computer-based text analysis not only for the identification of marker words associated with the specific coding categories of the Heckhausen system, but, building on earlier work by Stone and colleagues (1966), also defined rules for boundary conditions (e.g., words signifying failure should only be coded when followed by words signifying negative affect, but not when preceded by such words). The Seidenstückers' dictionary consisted of 59 categories covering a total of 920 words and its application to PSE stories yielded convergent validity coefficients of 0.53 for fear of failure and 0.78 for hope of success, the two main dimensions of the Heckhausen *n* Achievement coding system.

Pennebaker and King (1999) aimed to predict PSE motive scores based on Pennebaker and Francis's (1999) original version of the LIWC. This LIWC consisted of 72 dimensions of language use, which in turn covered more than 2200 words and word stems. It was designed to represent many broad and robust dimensions of language to be applicable to a diverse range of texts; it was not designed to capture marker words specifically related to implicit motives. Pennebaker and King collected PSEs from 79 participants and content-coded them for motive imagery and also LIWC-analyzed the stories. Using a four-factor solution for the LIWC dimensions derived in a previous study, they found that *n* Achievement and, to a lesser extent, *n* Affiliation correlated with LIWC factor scores, whereas *n* Power showed no reliable covariation with such scores. In addition, Pennebaker and King created a composite score of those LIWC factor scores that were predictive of the content-coding *n* Achievement score; the LIWC composite score converged at 0.44 with *n* Achievement. A drawback of this study is the fact that Pennebaker and King used LIWC factor scores instead of original LIWC category scores to explore convergent validity with content-coded motive scores. This not only makes the interpretation of the meaning of these factor scores difficult. It may also obscure instances of meaningful variance overlap between *specific* LIWC dimensions and content-coded motive scores, an effect that may have contributed to the low convergence for *n* Affiliation and the lack of convergence for *n* Power.

Finally, Hogenraad (2003, 2005) compiled dictionaries, in part based on earlier work with the General Inquirer system, that capture themes of power, achievement, and affiliation in historical and literary documents. He followed the lead of Winter (2010, for summary), who had shown with content-coding methods that international crises that escalate to aggressive encounters are preceded by an increase in power imagery and a decrease in affiliation imagery, whereas peaceful crisis resolutions are preceded by the opposite pattern in exchanges between the involved parties. Hogenraad validated his dictionaries through computer-based analysis of documents that reflect actual or fictional crises. Replicating Winter's (2010) findings, he demonstrated that crisis escalation is associated with an increase in power and a decrease in affiliation words, whereas crisis de-escalation is associated with an increase in affiliation and a decrease in power words. Thus, Hogenraad's dictionaries show similar criterion validity as Winter's applications of motive content-coding systems to historical documents. But it is unclear to what extent they capture the same underlying attributes, because Hogenraad did not report convergent validity coefficients between his dictionary-based text analyses and motive scores derived from content-coding of the same texts.

To summarize, there exists a veritable research tradition that has aimed at substituting, or at least supplementing, the laborious work of content-coding motivational themes by counting specific words. Some of this research has looked at word classes that are not a priori linked to a motive, such as the work by Pennebaker and King (1999). Other research has aimed at creating dictionaries that capture marker words specifically related to what a human coder would score as motive imagery (i.e., the work by Smith, 1968, Seidenstücker and Seidenstücker, 1974, and to some extent also Hogenraad, 2003, 2005). Much of this work has shown that examining word use in text can be a useful approximation for content-coded motive scores. So why didn't this assessment approach gain more traction, given its obvious advantages in terms of saving time and pushing coding objectivity to the max?

One reason is technology. The first such studies were conducted at a time when there were no computers around for processing data or doing statistics, and therefore little was gained in terms of speed or objectivity relative to content coding. Even when computers were finally available, they were slow, expensive, and cumbersome to work with. Thus, for a long time it was simply more efficient to content-code PSE stories by hand and run analyses with the scores derived in this manner. Clearly, this had changed when Pennebaker and King (1999) and Hogenraad (2003, 2005) took another stab at computer-based PSE scoring. Now computers are fast, user-friendly, and cheap. Standard experimental software allows presenting the PSE on the computer and harvesting the stories typed on the keyboard as ASCII files that can be further processed and analyzed (Schultheiss et al., 2008).

Another reason is generalizability. Smith (1968) pointed out that when marker-word dictionaries are derived for PSEs with a specific set of pictures, they may work well with stories collected with this particular PSE, but suffer considerable loss of validity when applied to stories collected with novel PSE pictures or when used with texts not generated by a PSE. This concern, although a valid one, was never systematically dealt with in subsequent research on computerized coding of PSEs for motive marker words. Pennebaker and King (1999) addressed this issue to some extent by showing in one of their studies that people's use of certain word categories stays remarkably stable across time and the situations in which text is produced. This may be the result of the LIWC authors' effort to make its word categories as comprehensive and broadly applicable as possible and thus to enhance generalizability. More specific dictionaries consisting only of few or narrowly defined motive marker words may not feature this advantage and Smith's (1968) warning may therefore remain valid.

A third reason is validation. Most previous studies focused on and reported evidence of convergent validity between computer-based and content-coding motive scores. In addition, Pennebaker and King (1999) also examined the predictive validity of computer-based scores, and Hogenraad's (2003, 2005) work focuses on criterion validity exclusively. But so far no research exists that has attempted a full-blown validation of the marker-word approach by showing that a computer-based word-count measure is (a) sensitive to causal manipulation of the motivational state, (b) predicts core criteria of motivation such as emotional responses to motive-related successes and failures, (c) converges with corresponding content-coding measures of the same motive, and (d) has discriminant validity vis-à-vis text-based measures of other motives and self-report measures of the same motivational domain (see McClelland, 1958, 1987). Such a validation would provide considerable support for the marker-word hypothesis and has the potential of advancing the assessment of implicit motives by making it completely objective and automatic in one stroke.

### The present research

The present research aims to overcome the limitations of earlier studies in several ways. First, I decided to use LIWC 2001 to analyze PSE stories, because the LIWC software and dictionaries are very accessible and are used by many researchers. The LIWC 2001 dictionary has been validated for both English (Pennebaker et al., 2001) and German texts (Wolf et al., 2008), as well as for several other languages. Its dictionary covers about 80% of the vocabulary that people use in everyday written and spoken language (Pennebaker et al., 2001). Moreover, the LIWC 2001 word categories were created with the goal of delineating useful classes of words for the description of formal aspects of language use (e.g., use of pronouns; past, present, future tense; use of punctuation), its functional aspects (e.g., words signifying causation, certainty, negation, temporal and spatial relations), reference to emotion (e.g., words signifying optimism, anxiety, anger), reference to fundamental perceptions and actions (e.g., words related to the senses) and reference to social categories and concerns (e.g., words related to school, friends, money, music). The LIWC 2001 word categories, and particularly its categories related to cognitive processes, emotion, and social life, thus represent a broad and diverse net through which important dimensions of language can be captured. This approach therefore also heeds Smith's (1968) warning that dictionaries should have sufficient generalizability. In contrast to Pennebaker and King's (1999) approach, however, I analyzed the utility of the LIWC dictionary at the level of the original categories, searching for linear combinations of categories that reliably converged with content-coded motive scores and whose weighted sum would maximize variance overlap with these scores. Thus, convergence of LIWC 2001 category scores with content-coded motive scores was the primary criterion for validation in this study.

To examine the robustness of the linear combinations of LIWC categories, I tested their *convergent validity* with content-coded motive scores in two different samples, one from the US and one from Germany, and thus in two different cultures and languages in Study 1. If the same marker-word categories predict motive scores in a similar way across these samples, they can be accorded a substantial degree of generalizability. I specifically expected content-coded *n* Achievement scores to converge with the LIWC *Achievement* category, because this category contains many words that would also be coded as achievement imagery with Winter's (1994) running text system (e.g., *award, efficient, excel, improve*). I expected content-coded *n* Power scores to converge with the LIWC *Anger* category, because it contains many words associated with strong, forceful actions, having control over others, or having negative emotional impact (e.g., *abuse, aggress, rape, annoyed, destroy, fight, insult, kill*), that is, words that have a high likelihood to occur in sentences that could be coded for *n* Power. Finally, I expected content-coded *n* Affiliation scores to converge with the LIWC *Social* category, and perhaps most closely with the subcategory *Friends*, which represents concepts and actions associated with what would be coded for *n* Affiliation (e.g., *friend, lover, soulmate, sweetheart*). Beyond these specific hypotheses, analyses were exploratory and focused on identifying replicable patterns of correlations between content-coded motive scores and LIWC 2001 categories.

In Study 1 I also explored whether LIWC-based scores show similar *criterion validity* as content-coded scores by examining their ability (a) to reproduce a gender effect frequently observed for *n* Affiliation (see Duncan and Peterson, 2010) and (b) to predict, in interaction with self-reported goal progress, emotional well-being, another effect frequently observed for content-coding motive measures (see Hofer and Busch, 2013, for a review). LIWC-based scores' *discriminant validity* was tested by examining the degree to which scores for *n* Affiliation, *n* Achievement, and *n* Power are (a) independent from each other and (b) independent from self-report measures of motivational needs. Finally, in Study 2 I tested whether LIWC-based motive scores have causal validity, that is, whether experimental manipulation of motivational states leads to corresponding changes in PSE stories (see McClelland, 1958; Borsboom et al., 2004).

## Study 1: deriving and validating LIWC-based motive scores in us and german samples

To test the marker-word hypothesis and to derive robust linear combinations of LIWC 2001 word categories that converge with content-coded motive scores, I reanalyzed PSEs collected in a research project dealing with the congruence between implicit and explicit motives (Studies 2 and 3 in Schultheiss et al., 2011). I also included activity inhibition, the frequency with which the negation *not* (German: *nicht*) occurs in the PSE, in the analysis. This variable represents a propensity to engage the right hemisphere in response to stress and is frequently assessed along with implicit motive measures, because it often moderates the effects of motives on outcome measures (see Schultheiss et al., 2009; Langens, 2010).

### Method

#### US sample

One-hundred-and-forty-six students at the University of Michigan, Ann Arbor, USA, participated in a cross-sectional study on “attention and performance” for course credit. Of these, 113 participants (average age: 19 years; 57 mens, 48 womens, 8 participants did not give demographic information) provided a complete PSE and were included in the analyses. Of the participants who had provided demographic information, 62% self-identified as Caucasian, 20% as Asian, 6% as African-American, 3% as Pacific Islander; the remainder belonged to other or mixed ethnic groups.

#### German sample

One-hundred-six students at Friedrich-Alexander University, Erlangen, Germany, participated in a cross-sectional study on “attention and performance” for payment of €15. Of the initial sample, 100 participants (average age: 23 years; 51 mens; all Caucasian) provided a complete PSE.

#### PSE

US participants worked on an 8-picture PSE described by Schultheiss et al. (2008), and German participants completed the 6-picture PSE by Pang and Schultheiss (2005), which does not contain the pictures *bicycle race* and *girlfriends in café* of the former PSE. Thus, US and German participants' PSEs were identical for 6 pictures. All participants followed standard instructions for PSE computer administration described in Schultheiss and Pang (2007). The PSE was programmed in (Inquisit, 2005) (Millisecond Software, Seattle, WA). Picture order was randomized for each participant. Each picture was shown for 10 s. Participants were instructed to type their stories directly into a window on the screen. After 4 min had elapsed, participants were prompted to finish the story and move on to the next picture.

Stories were later coded for motivational imagery by trained scorers using Winter's (1994) running-text system. All scorers had previously exceeded 85% inter-scorer agreement on calibration materials prescored by an expert. A single coder scored PSE stories in the US sample; two coders independently scored all PSE stories in the German sample. Their scores were averaged for all further analyses. Their interrater reliabilities (Pearson's *r*) for *n* Power, *n* Achievement and *n* Affiliation were 0.79, 0.74, and 0.86. Raw motive and activity inhibition scores (all square-root transformed in the US sample to correct for distribution skew) were correlated significantly with PSE total word count (*r*s = 0.30 −0.53, *p*s < 0.005) and were therefore residualized for PSE total word count. Residuals were converted to *z* scores for all further inferential analyses.

#### LIWC 2001

All PSE stories were first spell-checked and corrected and then individually saved and run through the LIWC software (http://www.liwc.net/). For the US sample, I used the original English dictionary developed and validated by Pennebaker et al. (2001). For the German sample, I used the German dictionary translation validated by Wolf et al. (2008). Both dictionaries are comprised of the same 74 categories. These include standard linguistic dimensions (e.g., *Word count, Articles, Prepositions*), psychological processes related to affect and emotion (e.g., *Positive feelings, Anxiety*), cognition (e.g., *Causation, Insight, Certainty*), sensing and perceiving (*See, Hear, Feel*), and the social world (e.g., *Communication, Friends, Family*), words signifying relativity (e.g., verb tenses, *Space*, and *Motion*), and words related to personal concerns (e.g., *School, Achievement, Money, Sex, Eating*). In addition, information about punctuation (e.g., *Exclamation mark, Question mark, Quotation*) was also extracted. See Table 1 for a complete list of all variables and examples for each category. Except for *Word count* and *Words per sentence*, all LIWC categories are expressed as percentage scores to represent the number of target words relative to total words.

**Table 1:** **Descriptive statistics (*M, SD*), variance accounted for by picture cues (η^2^_Picture_), and internal consistency estimates across picture stories (Cronbach's alpha) for German (6-picture PSE) and US samples (6- and 8-picture PSEs) for content-coded raw motive scores and LIWC 2001 categories in Study 1**.

	**LIWC 2001 category examples (from Pennebaker et al., 2001**	**German sample**				**US sample**							
						**6-picture PSE**				**8-picture PSE**			
		**η^2^_Picture_**	**Alpha**	***M***	***SD***	**η^2^_Picture_**	**Alpha**	***M***	***SD***	**η^2^_Picture_**	**Alpha**	***M***	***SD***
**PSE**													
*n* Power		0.104^***^	0.36	4.55	2.36	0.058^***^	0.61	3.79	2.89	0.052^***^	0.59	5.43	3.41
*n* Achievement		0.534^***^	0.51	5.14	2.52	0.498^***^	0.55	4.56	2.55	0.577^***^	0.58	6.61	3.00
*n* Affiliation		0.610^***^	0.48	6.61	2.74	0.551^*^	0.50	4.91	2.65	0.565^***^	0.52	7.04	3.14
Activity inhibition		0.023^*^	0.45	5.58	3.57	0.012	0.64	5.00	3.90	0.017	0.65	6.92	4.67
**LIWC 2001**													
Word count		0.054^***^	0.93	607.85	151.54	0.001	0.94	668.09	206.15	0.023^*^	0.95	901.91	272.5
Words/ sentence		0.020	0.76	15.04	3.46	0.058^***^	0.78	17.31	3.62	0.048^***^	0.82	17.49	3.48
Unique words		0.027^*^	0.80	75.39	4.27	0.092^***^	0.85	64.32	6.04	0.079^***^	0.89	64.31	5.80
Dictionary words		0.168^***^	0.58	70.37	3.23	0.210^***^	0.73	77.68	3.85	0.275^***^	0.78	78.01	3.71
Words > 6 letters		0.204^***^	0.61	22.35	2.85	0.297^***^	0.64	15.63	2.72	0.272^***^	0.69	15.12	2.54
Pronouns	I, our, they, you're	0.092^***^	0.67	10.54	2.49	0.082^***^	0.67	10.22	2.34	0.090^***^	0.74	10.28	2.27
I	I, my, me	0.018	0.60	0.52	0.96	0.014	0.75	0.29	0.89	0.017	0.80	0.28	0.84
We	We, our, us	0.019	0.49	0.20	0.44	0.020^*^	0.14	0.06	0.13	0.017	0.45	0.06	0.17
Self	I, we, me	0.015	0.68	0.72	1.26	0.018	0.73	0.35	0.94	0.023^*^	0.79	0.35	0.91
You	You, you'll	0.318^***^	0.28	1.52	0.66	0.015	0.81	0.16	0.56	0.019^*^	0.83	0.14	0.48
Other	She, their, them	0.097^***^	0.63	9.42	2.29	0.127^***^	0.63	8.65	2.11	0.121^***^	0.71	8.74	2.06
Negate	No, never, not	0.032^**^	0.35	1.37	0.65	0.039^***^	0.61	0.95	0.63	0.031^***^	0.66	0.94	0.57
Assent	Yes, OK	0.012	0.35	0.20	0.25	0.055^***^	0.39	0.09	0.16	0.044^***^	0.52	0.08	0.15
Article	A, an, the	0.164^***^	0.70	10.65	2.24	0.169^***^	0.73	9.94	2.25	0.173^***^	0.77	9.76	2.10
Prepositions	On, to, from	0.096^***^	0.58	9.55	1.65	0.018	0.48	13.63	1.57	0.017	0.56	13.73	1.51
Number	One, thirty, million	0.017	0.08	0.42	0.31	0.047^***^	0.32	1.08	0.55	0.113^***^	0.39	1.28	0.56
Affect	Happy, ugly, bitter	0.123^***^	0.48	5.65	1.52	0.143^***^	0.33	4.71	1.18	0.119^***^	0.38	4.60	1.03
Positive emotions	Happy, pretty, good	0.110^***^	0.50	3.81	1.26	0.286^***^	0.40	3.34	1.07	0.242^***^	0.48	3.25	0.96
Positive feelings	Happy, joy, love	0.117^***^	0.21	0.59	0.40	0.321^***^	0.12	0.91	0.50	0.292^***^	0.24	0.86	0.44
Optimism	Certainty, pride, win	0.101^***^	0.35	0.92	0.49	0.205^***^	0.22	0.81	0.45	0.276^***^	0.32	0.90	0.43
Negative emotions	Hate, worthless, enemy	0.200^***^	0.29	1.84	0.83	0.196^***^	0.15	1.35	0.63	0.161^***^	0.23	1.33	0.56
Anxiety	Nervous, afraid, tense	0.045^***^	−0.13	0.30	0.24	0.072^***^	0.44	0.41	0.38	0.070^***^	0.42	0.37	0.31
Anger	Hate, kill, pissed	0.288^***^	0.11	0.45	0.37	0.242^***^	0.01	0.46	0.34	0.207^***^	0.14	0.42	0.30
Sadness	Grief, cry, sad	0.030^**^	0.13	0.41	0.34	0.020^*^	0.16	0.24	0.24	0.015	0.02	0.24	0.19
Cognitive processes	Cause, know, ought	0.119^***^	0.46	8.67	1.83	0.092^***^	0.28	5.55	1.23	0.074^***^	0.40	5.61	1.14
Causation	Because, effect, hence	0.017	0.06	1.21	0.49	0.060^***^	0.18	0.75	0.41	0.051^***^	0.35	0.73	0.38
Insight	Think, know, consider	0.088^***^	0.49	2.44	0.95	0.052^***^	0.14	1.77	0.63	0.048^***^	0.21	1.79	0.56
Discrepancy	Should, would, could	0.024^*^	0.43	1.71	0.71	0.022^*^	0.25	1.64	0.65	0.023^*^	0.38	1.66	0.61
Inhibition	Block, constrain	0.037^***^	0.08	0.22	0.22	0.016	−0.02	0.30	0.24	0.015	0.04	0.30	0.21
Tentative	Maybe, perhaps, guess	0.040^***^	0.68	1.21	0.9	0.021^*^	0.79	1.58	1.21	0.046^***^	0.83	1.54	1.13
Certainty	Always, never	0.124^***^	0.42	2.06	0.83	0.039^***^	0.53	1.03	0.62	0.040^***^	0.62	1.02	0.59
Senses	See, touch, listen	0.146^***^	0.01	0.25	0.22	0.079^***^	0.49	2.23	0.94	0.219^***^	0.61	2.36	0.91
See	View, saw, look	0.069^***^	−0.06	0.12	0.14	0.022^*^	0.33	0.95	0.55	0.040^***^	0.47	0.94	0.52
Hear	Heard, listen, sound	NC	0.20	0.03	0.08	0.256^***^	0.35	0.78	0.5	0.342^***^	0.45	0.89	0.50
Feel	Touch, hold, felt	NC	−0.02	0.01	0.03	0.046^***^	0.48	0.40	0.40	0.041^***^	0.50	0.40	0.34
Social	Talk, us, friend	0.096^***^	0.60	13.59	2.47	0.091^***^	0.57	13.73	2.55	0.323^***^	0.64	14.29	2.46
Communication	Talk, share, converse	0.250^***^	0.24	1.32	0.59	0.263^***^	0.22	1.47	0.62	0.396^***^	0.36	1.63	0.63
Other references	1st pl, 2nd, 3rd per prns	0.082^***^	0.62	10.33	2.24	0.116^**^	0.62	9.01	2.06	0.117^***^	0.70	9.09	2.03
Friends	Pal, buddy, coworker	0.026^*^	−0.30	0.48	0.29	0.026^*^	0.25	0.28	0.30	0.227^***^	0.21	0.43	0.36
Family	Mom, brother, cousin	0.044^***^	0.61	1.20	1.01	0.003	0.33	0.39	0.51	0.013	0.22	0.39	0.45
Humans	Boy, woman, group	0.071^***^	0.67	1.51	1.18	0.099^***^	0.71	2.14	1.41	0.142^***^	0.79	2.25	1.43
Time	Hour, day, oclock	0.052^***^	0.56	5.36	1.65	0.152^***^	0.46	4.13	1.22	0.116^***^	0.50	4.22	1.10
Past	Walked, were, had	0.018	0.90	4.23	3.12	0.047^***^	0.91	3.55	3.13	0.040^***^	0.93	3.60	3.17
Present	Walk, is, be	0.068^***^	0.87	6.20	2.83	0.045^***^	0.86	10.54	3.52	0.039^***^	0.90	10.65	3.44
Future	Will, might, shall	0.015	0.63	0.78	0.65	0.020^*^	0.70	1.47	1.06	0.025^**^	0.74	1.40	0.95
Space	Around, over, up	0.079^***^	0.50	7.78	1.49	0.068^***^	0.37	3.32	0.94	0.077^***^	0.41	3.47	0.85
Up	Up, above, over	0.080^***^	0.45	1.57	0.71	0.044^***^	0.15	1.54	0.56	0.041^***^	0.29	1.61	0.55
Down	Down, below, under	0.021	0.08	0.10	0.18	0.055^***^	−0.10	0.25	0.23	0.045^***^	0.04	0.25	0.20
Inclusive	With, and, include	0.105^***^	0.54	6.32	1.29	0.096^***^	0.53	7.30	1.45	0.073^***^	0.55	7.29	1.26
Exclusive	But, except, without	0.024^*^	0.43	2.15	0.74	0.003	0.55	2.76	1.00	0.002	0.59	2.76	0.89
Motion	Walk, move, go	0.069^***^	0.31	1.25	0.54	0.091^***^	0.34	1.36	0.61	0.069^***^	0.27	1.32	0.50
Occupation	Work, class, boss	0.468^***^	0.25	4.38	1.21	0.358^***^	−0.09	2.64	0.84	0.335^***^	0.01	2.68	0.75
School	Class, student, college	0.379^***^	0.09	1.42	0.69	0.274^***^	−0.11	0.72	0.61	0.255^***^	−0.07	0.63	0.49
Job	Employ, boss, career	0.369^***^	0.05	1.46	0.57	0.121^***^	0.13	0.89	0.51	0.130^***^	0.10	0.78	0.40
Achievement	Try, goal, win	0.245^***^	0.32	2.22	0.86	0.182^***^	−0.03	1.33	0.59	0.320^***^	0.14	1.54	0.57
Leisure	House, TV, music	0.406^***^	−0.12	1.36	0.51	0.511^***^	−0.11	1.22	0.52	0.470^***^	−0.09	1.08	0.42
Home	House, kitchen, lawn	0.032^*^	0.39	0.34	0.35	0.030^***^	0.28	0.32	0.31	0.025^**^	0.22	0.30	0.26
Sports	Football, game, play	0.434^***^	0.03	0.55	0.38	0.141^***^	−0.07	0.24	0.22	0.137^***^	0.06	0.25	0.21
TV	TV, sitcom, cinema	0.086^***^	0.15	0.13	0.18	0.097^***^	0.10	0.13	0.18	0.099^***^	0.10	0.11	0.14
Music	Tunes, song, cd	0.506^***^	−0.18	0.46	0.27	0.616^***^	−0.06	0.43	0.29	NC	−0.06	0.33	0.22
Money	cash, taxes, income	0.074^***^	−0.22	0.52	0.33	0.143^***^	0.15	0.39	0.36	0.149^***^	0.30	0.33	0.31
Metaphysical issues	God, heaven, coffin	0.040^***^	0.29	0.37	0.32	0.007	0.06	0.11	0.15	0.008	0.21	0.10	0.13
Religion	God, church, rabbi	0.045^***^	0.25	0.27	0.26	0.011	0.04	0.04	0.09	0.008	0.26	0.04	0.09
Death	Dead, burial, coffin	0.012	0.19	0.14	0.22	0.018	0.05	0.07	0.12	0.019^*^	0.23	0.06	0.10
Physical states	Ache, breast, sleep	0.189^***^	0.23	1.26	0.55	0.232^***^	0.04	1.42	0.60	0.190^***^	0.19	1.54	0.59
Body	Ache, heart, cough	0.092^***^	0.47	0.65	0.48	0.051^***^	0.16	0.60	0.43	0.073^***^	0.24	0.67	0.42
Sexuality	Lust, penis, fuck	0.083^***^	0.07	0.18	0.20	0.074^***^	0.18	0.31	0.31	0.064^***^	0.27	0.28	0.28
Eating	Eat, swallow, taste	0.308^***^	−0.06	0.38	0.29	0.411^***^	0.16	0.45	0.35	0.377^***^	0.26	0.52	0.34
Sleeping	Asleep, bed, dreams	0.019	0.15	0.07	0.15	0.024^*^	0.16	0.10	0.16	0.058^***^	0.19	0.12	0.14
Grooming	Wash, bath, clean	0.008	−0.15	0.04	0.08	0.011	0.00	0.03	0.09	0.057^***^	0.13	0.07	0.15
Swear words	Damn, fuck, piss	0.012	0.57	0.04	0.12	0.014	0.40	0.02	0.09	0.013	0.71	0.02	0.10
Fillers	You know, I mean	NC	0.11	0.01	0.05	0.003	0.19	0.15	0.19	0.003	0.25	0.15	0.16
Question mark	?	0.018	0.56	0.20	0.32	0.012	0.70	0.07	0.27	0.012	0.77	0.07	0.26
Exclamation mark	!	0.013	0.88	0.30	0.82	0.020^*^	0.38	0.08	0.22	0.017	0.52	0.09	0.22
Quote	“”	0.023^*^	0.76	0.41	0.84	0.017	0.74	0.34	0.98	0.017	0.84	0.36	1.02
All punctuation	.;:!?	0.019	0.88	14.14	3.84	0.019	0.88	11.07	3.81	0.015	0.92	11.11	3.79

NC, not computed, because one picture story or more had no variance on this variable.

* *p* < 0.05,

** *p* < 0.01,

*** *p* < 0.005.

#### PRF

I used scales from the PRF (English version: (Jackson, 1984); German version: Stumpf et al. (1985) to assess participants' self-attributed needs for affiliation, achievement, and power. The first two needs were measured with the correspondingly named PRF scales; the last was assessed through the scales *dominance* and *aggression*, because both capture self-attributed behaviors that are consistent with the definition and coding of *n* Power at the implicit level. Each PRF scale includes 16 True/False (1/0) statements that describe values, habits, and preferences consistent or inconsistent with each motivational need. Participants were asked to decide how representative each statement was as a self-description.

#### Criterion validity measures

In addition to their gender, participants in the German sample also reported their current progress on ideographically assessed personal goals related to achievement and power on Brunstein et al.'s (1998) 4-item *goal progress* scale. Cronbach's alpha, mean, and standard deviation were 0.85, 14.54, and 3.17 for the achievement-progress scale and 0.90, 13.23, and 3.76 for the power-goal progress scale, respectively. Emotional well-being was assessed with the 8-item *hedonic tone* scale of Matthews et al.'s (1990) mood adjective check list (Cronbach's alpha = 0.91, M = 22.95, *SD* = 5.27).

### Results and discussion

#### Picture profiles and descriptive statistics

Table 1 provides descriptive statistics, internal consistency estimates across pictures, and estimates of the proportion of total score variance accounted for by the pictures for PSE raw motive scores, activity inhibition, and all LIWC categories. As in previous studies (e.g., Schultheiss and Brunstein, 2001; Pang and Schultheiss, 2005), picture cues accounted for sizeable proportions of variance in motive imagery. Picture effects were particularly pronounced for *n* Achievement and *n* Affiliation, accounting for about half of the variance, but less so for *n* Power and activity inhibition, which tended to be expressed more evenly across pictures. Internal consistency coefficients (Cronbach's alpha) for motive imagery scores reached levels acceptable for scientific studies (i.e., 0.60). However, these levels may reflect to a considerable extent an effect of word count, which is highly consistent across pictures and which is positively correlated with the presence of motivational imagery in the stories (for a discussion of the relevance of internal consistency in the context of implicit motive assessment, see Schultheiss et al., 2008).

Although the LIWC scores reported in Table 1 are primarily intended to provide descriptive detail about the PSE as viewed through the lens of the LIWC 2001 dictionary categories and will not be discussed in detail, a few general observations can be made. First, comparing the overall frequency patterns of word use in the present study with the frequencies reported for the LIWC 2001 dictionary (Pennebaker et al., 2001), the most striking difference is the almost complete lack of first-person language on the PSE (*I*, *We, Self*) and the concomitant emphasis on third-person language (*Other*). Thus, unlike the samples of self-referential writing and free speech that Pennebaker et al. (2001) analyzed, PSE writers almost never take the first-person perspective and almost always prefer a third-person narrative (see also Turk et al., 2010). If the first person is used at all, it is often in the context of dialog (*Self* correlates with the use of quotation marks at 0.38 in the US and at 0.20 in the German sample, *p*s < 0.05). In a sense, then, PSE narrators almost never explicitly talk about themselves and almost always about other people, an effect that is consistent with the PSE's instructions and with the broader intent behind this mode of personality assessment (see Morgan and Murray, 1935; McClelland, 1980). No other category diverges as strongly from the descriptive data provided by Pennebaker et al. (2001).

A second point worth making is that LIWC analysis of the PSE allows to partition the variance in language that is due to the eliciting cues (i.e., the PSE pictures, represented by η^2^_Picture_ in Table 1) and the variance that is due to stable individual differences (represented by Cronbach's alpha in Table 1). Not surprisingly, LIWC categories that are infrequent (i.e., that have a mean close to 0) show little internal consistency across pictures and often do not vary much by picture (e.g., *Number* and *Metaphysical Issues*), although some of the low-frequency categories can pick up strong picture effects (e.g., *Senses* or *Leisure*). In contrast, many high-frequency LIWC categories consistently pick up pervasive effects of both picture differences and individual differences. For instance, in both samples the use of articles varies markedly and significantly by picture, but stable individual differences are also in evidence, as reflected in Cronbach alphas ≥0.70. A combination of marked picture differences and individual differences seems to characterize stylistic LIWC categories, like *Pronouns, W*ords >6 Letters, verb tense, or common words (as reflected by their coverage of the LIWC 2001 dictionary) in both samples. In contrast, the use of words in the categories *Positive Emotions* and *Negative Emotions, Communication, Occupation, Leisure*, and *Physical State* appears to be more strongly influenced by the pictures, with variance proportions ranging from 11 to 51%, than by stable individual dispositions (αs < 0.50).

A third feature is the scarcity with which many categories (particularly subcategories) show up in the stories. Many category scores therefore deviate substantially from a normal distribution, violating an important prerequisite for regression-based inferential statistics. For all further analyses, I resolved deviations from normality, as revealed through significant Kolmogorov-Smirnov tests and inspection of score distribution histograms, as follows: (1) if the score distribution was sufficiently differentiated at the low scale end in both samples, I subjected it to a log transformation [new score = ln (1 + old score)] for both samples; (2) if a large proportion of participants (i.e., >20%) had a score of 0 in one or both samples, I converted the variable to a dichotomous format, with 0 representing the absence of the use of words in a category and 1 the presence of such words. Table 2 indicates the transformations that were used.

**Table 2:** **Correlations between content-coded *n* Power (*n* Pow), *n* Achievement (*n* Ach), *n* Affiliation (*n* Aff), and activity inhibition (AI) scores (all residualized for PSE protocol length) and LIWC 2001 categories for German (6-picture PSE) and US samples (6- and 8-picture PSEs) in Study 1**.

		**German sample**				**US sample**							
						**6-picture PSE**				**8-picture PSE**			
**LIWC 2001 category**	**Transformation**	***n* Pow**	***n* Ach**	***n* Aff**	**AI**	***n* Pow**	***n* Ach**	***n* Aff**	**AI**	***n* Pow**	***n* Ach**	***n* Aff**	**AI**
Word count		0.00	0.00	0.00	0.00	0.00	0.00	0.00	0.00	0.00	0.00	0.00	0.00
Words/ sentence		0.02	−0.12	−0.13	−0.10	0.06	0.02	−0.07	−0.28^**^	0.07	−0.02	−0.04	−0.32^***^
Unique words		0.17	−0.01	−0.13	−0.15	0.02	0.03	−0.10	0.06	0.00	−0.01	−0.10	0.09
Dictionary words		−0.27^**^	0.09	0.19	0.02	0.01	−0.11	0.17	0.15	0.03	−0.08	0.18	0.09
Words > 6 letters		0.12	0.06	−0.21^*^	−0.33^***^	−0.13	0.05	−0.15	−0.25^**^	−0.17	0.01	−0.15	−0.23^***^
Pronouns		0.09	0.06	0.15	0.29^***^	0.01	−0.04	0.14	0.35^***^	−0.05	−0.02	0.14	0.40^***^
I	Dichotomized	0.08	−0.02	−0.05	0.19	−0.02	−0.22^*^	−0.07	0.13	0.00	−0.09	−0.17	0.05
We	Dichotomized	−0.01	−0.07	−0.06	0.08	0.07	−0.23^*^	−0.12	0.13	0.14	−0.23^*^	−0.12	0.12
Self	Dichotomized	0.00	−0.01	−0.02	0.10	0.01	−0.36^***^	−0.10	0.14	0.07	−0.28^***^	−0.15	0.05
You	Logarithm	0.13	0.16	−0.04	−0.05	0.01	−0.10	0.03	0.00	−0.05	−0.07	−0.02	0.11
Other		0.07	0.08	0.30^***^	0.18	0.10	0.12	0.37^***^	0.19	0.03	0.10	0.40^***^	0.26^***^
Negate		−0.01	−0.34^***^	−0.15	0.85^***^	0.04	−0.18	−0.07	0.69^***^	0.01	−0.20^*^	−0.12	0.66^***^
Assent	Dichotomized	−0.03	0.14	−0.07	0.30^***^	0.02	−0.06	−0.05	0.01	0.09	−0.06	−0.04	0.08
Article		−0.24^*^	−0.02	−0.12	−0.22^*^	0.03	−0.02	−0.03	−0.25^**^	0.11	−0.02	−0.02	−0.31^***^
Prepositions		0.14	−0.01	−0.12	−0.34^***^	0.05	0.00	0.01	−0.10	0.02	0.05	0.02	−0.21^*^
Number		0.09	−0.03	0.06	−0.14	0.09	0.06	−0.03	−0.07	0.07	0.10	0.05	−0.15
Affect		0.00	0.19	0.18	−0.06	−0.05	0.12	0.05	0.11	−0.04	0.10	0.05	0.11
Positive emotions		−0.14	0.18	0.26^**^	−0.06	−0.15	0.21^*^	0.15	−0.03	−0.13	0.21^*^	0.16	−0.07
Positive feelings	Logarithm	−0.17	−0.03	0.33^***^	0.11	−0.11	−0.02	0.26^**^	−0.05	−0.09	−0.05	0.23^*^	−0.10
Optimism		0.06	0.31^***^	0.12	−0.16	−0.07	0.30^***^	0.31^***^	−0.03	−0.04	0.33^***^	0.28^***^	−0.12
Negative emotions		0.21^*^	0.07	−0.06	−0.01	0.16	−0.14	−0.16	0.23	0.14	−0.17	−0.18^*^	0.32^***^
Anxiety	Logarithm	0.00	0.26^*^	0.15	−0.13	0.01	−0.06	0.05	0.02	0.01	−0.11	0.04	0.08
Anger	Logarithm	0.21^*^	0.10	−0.04	−0.13	0.24^**^	−0.17	−0.19^*^	0.14	0.25^**^	−0.13	−0.19^*^	0.24^***^
Sadness	Logarithm	0.12	−0.04	0.06	−0.01	−0.09	0.17	−0.06	0.18	−0.01	0.11	−0.03	0.18
Cognitive processes		−0.20^*^	−0.13	−0.05	0.37^***^	0.02	0.01	−0.03	0.19^*^	0.03	0.00	0.01	0.18^*^
Causation		0.10	−0.17	−0.01	0.26^*^	0.03	−0.06	−0.07	−0.05	−0.06	−0.03	−0.02	−0.01
Insight		−0.18	−0.25^*^	−0.05	0.11	0.09	0.10	0.03	0.00	0.08	0.09	−0.02	0.02
Discrepancy		−0.00	0.04	−0.10	0.31^***^	−0.07	0.02	0.09	0.26^**^	−0.04	0.02	0.16	0.29^**^
Inhibition	Dichotomized	0.09	−0.03	−0.10	0.09	0.09	−0.07	0.00	0.13	0.04	−0.09	0.00	0.10
Tentative	Logarithm	−0.40^***^	−0.24^*^	−0.15	0.25^*^	−0.28^***^	−0.23^*^	−0.21^*^	0.05	−0.22^*^	−0.27^***^	−0.27^***^	−0.01
Certainty		−0.02	0.11	0.13	0.02	0.01	0.18	0.06	−0.02	−0.03	0.16	0.04	0.00
Senses	Dichotomized	0.02	0.11	0.09	−0.29^***^	−0.02	−0.13	0.01	0.09	0.00	−0.13	0.02	0.10
See	Dichotomized	0.27^**^	0.28^***^	0.13	−0.28^***^	−0.09	−0.06	0.00	−0.06	0.00	−0.07	−0.03	−0.05
Hear	Dichotomized	−0.20^*^	−0.12	0.13	0.06	0.05	−0.07	0.01	0.24^*^	0.03	−0.07	0.02	0.23^*^
Feel	Dichotomized	0.14	0.02	−0.16	0.01	0.01	−0.02	0.00	0.05	−0.01	−0.08	0.07	0.02
Social		−0.04	−0.06	0.37^***^	0.23^*^	0.10	−0.03	0.26^**^	0.20^*^	0.06	−0.02	0.27^***^	0.20^*^
Communication		−0.15	−0.05	0.28^**^	0.15	0.08	−0.09	−0.08	0.03	0.05	−0.02	−0.05	0.04
Other references		0.10	0.12	0.25^*^	0.22^*^	0.08	0.07	0.31^***^	0.25^**^	0.01	0.07	0.33^***^	0.31^***^
Friends	Logarithm	0.02	−0.09	0.28^**^	0.00	0.14	−0.02	0.37^***^	0.04	0.08	−0.10	0.36^***^	0.04
Family	Logarithm	−0.14	−0.22^*^	0.15	0.00	0.00	−0.11	−0.03	0.01	−0.05	−0.17	−0.02	−0.02
Humans	Logarithm	−0.23^*^	−0.22^*^	0.09	0.03	0.01	−0.05	−0.04	0.00	0.06	−0.05	−0.06	−0.08
Time		0.09	0.15	0.03	−0.25^*^	−0.10	0.00	0.10	−0.07	−0.13	−0.01	0.14	−0.02
Past	Logarithm	0.14	0.09	−0.17	0.06	0.04	0.07	−0.08	0.08	−0.03	0.07	−0.04	0.14
Present		−0.17	−0.03	0.18	0.11	0.02	−0.10	0.19^*^	0.03	0.10	−0.07	0.11	0.01
Future	Logarithm	−0.09	−0.02	0.22^*^	−0.09	−0.21^*^	−0.03	0.05	−0.02	−0.18	−0.07	0.01	−0.06
Space		0.09	−0.06	−0.05	−0.36^***^	0.27^***^	−0.07	0.06	0.11	0.20^*^	−0.02	0.02	0.13
Up		−0.05	0.08	0.11	−0.16	0.12	−0.13	0.03	0.08	0.07	−0.12	−0.07	0.08
Down	Dichotomized	0.04	−0.02	−0.08	0.12	0.22^*^	0.10	−0.04	0.08	0.23^*^	0.10	−0.07	0.11
Inclusive		−0.03	0.00	0.02	−0.20^*^	0.03	−0.04	0.09	−0.19^*^	0.03	−0.03	0.09	−0.20^*^
Exclusive		0.13	−0.07	−0.01	0.10	0.16	0.00	−0.02	0.22^*^	0.15	0.02	−0.01	0.21^*^
Motion		0.00	0.14	0.07	−0.24^*^	0.01	−0.15	0.09	−0.06	0.01	−0.12	0.05	−0.01
Occupation		0.00	0.22^*^	0.05	−0.20^*^	0.01	0.25^**^	0.08	−0.07	−0.07	0.31^***^	0.08	−0.14
School	Logarithm	0.05	0.22^*^	−0.10	−0.17	−0.07	−0.07	0.02	−0.19^*^	−0.09	−0.03	0.02	−0.14
Job		0.02	0.00	0.07	−0.12	0.02	0.12	0.02	0.15	0.00	0.14	0.05	0.07
Achievement	Logarithm	−0.05	0.21^*^	0.08	−0.10	0.00	0.35^***^	0.11	0.06	−0.08	0.36^***^	0.06	−0.08
Leisure		−0.13	0.08	0.07	−0.17	−0.02	−0.04	0.09	−0.06	−0.01	−0.08	0.02	−0.04
Home	Logarithm	0.08	−0.06	−0.01	−0.21^*^	−0.10	0.07	0.18	−0.28^***^	−0.15	0.05	0.15	−0.26^***^
Sports	Logarithm	−0.15	0.18	−0.02	−0.12	−0.02	−0.12	0.00	0.17	0.00	−0.09	−0.13	0.12
TV	Dichotomized	0.12	0.20^*^	0.01	−0.16	0.08	0.13	0.02	−0.10	0.06	0.02	−0.02	−0.01
Music		−0.20^*^	0.06	0.06	−0.08	−0.06	−0.11	−0.03	0.07	−0.01	−0.16	−0.05	0.15
Money	Logarithm	0.05	−0.09	−0.09	0.05	0.18	0.06	−0.14	0.02	0.23^*^	0.11	−0.09	0.10
Metaphysical issues	Dichotomized	0.00	0.08	−0.01	−0.07	0.09	0.24^*^	0.08	0.08	0.12	0.28^***^	0.05	0.15
Religion	Dichotomized	0.05	0.01	−0.02	−0.02	−0.16	0.06	0.10	0.08	−0.12	0.11	0.06	0.06
Death	Dichotomized	0.13	−0.03	−0.03	−0.10	0.14	0.22^*^	0.03	0.01	0.14	0.25^**^	0.03	0.08
Physical states		−0.13	0.04	−0.01	−0.16	−0.09	−0.14	0.17	−0.10	−0.01	−0.09	0.14	−0.03
Body	Logarithm	0.01	−0.01	−0.09	−0.04	−0.10	−0.10	−0.04	−0.04	−0.01	−0.06	−0.11	0.04
Sexuality	Dichotomized	−0.02	0.01	0.19	0.03	−0.00	0.02	0.35^***^	0.03	0.05	0.02	0.29^**^	0.07
Eating	Logarithm	−0.22^*^	0.11	0.00	−0.16	0.04	−0.12	0.07	−0.12	−0.02	−0.10	0.15	−0.14
Sleeping	Dichotomized	−0.05	0.04	0.05	−0.10	−0.15	−0.06	0.03	0.00	−0.09	0.02	0.16	−0.05
Grooming	Dichotomized	0.17	−0.07	0.01	−0.16	0.07	0.10	−0.08	−0.10	0.02	−0.11	−0.10	−0.04
Swear words	Dichotomized	0.05	−0.09	−0.07	0.12	−0.02	−0.06	0.08	0.23^*^	−0.04	−0.03	−0.07	0.28^***^
Fillers	Dichotomized	0.00	−0.06	−0.11	0.01	−0.09	−0.14	−0.01	−0.01	−0.09	−0.16	−0.03	−0.02
Question mark	Dichotomized	0.00	−0.22^*^	−0.06	0.34^***^	−0.01	−0.04	−0.19^*^	0.12	0.01	0.01	−0.21^*^	0.22^*^
Exclamation mark	Dichotomized	0.25^*^	0.02	−0.12	0.11	0.01	−0.14	−0.01	0.23^*^	0.07	−0.10	−0.06	0.22^*^
Quote	Dichotomized	−0.03	−0.17	−0.12	0.25^*^	0.02	−0.04	−0.09	0.04	−0.01	−0.06	−0.19^*^	0.06
All punctuation	Logarithm	0.13	−0.07	−0.05	0.32^***^	−0.05	−0.06	−0.23^*^	0.27^***^	−0.08	−0.05	−0.28^***^	0.26^***^

* *p* < 0.05,

** *p* < 0.01,

*** *p* < 0.005.

#### Convergent validity

Table 2 shows zero-order correlations between implicit motive scores and LIWC categories for the German and the US sample. The findings for the 6-picture PSE suggest that each motive was associated with two or more LIWC categories in a replicable fashion across samples. Across both samples and supporting predictions, *n* Power was positively associated with *Anger, n* Achievement with *Achievement*, but also with *Positive Emotions, Optimism*, and *Occupation*, and *n* Affiliation was positively associated with *Social* and *Friends*, but also with third-person pronouns (*Other References*) and *Positive Feelings*. Other word categories were clearly associated with motive scores in one sample and with a similar effect size, but below the significance threshold in the other. This was the case for the association between *n* Achievement and *Negations* and *Family* (both negative) and *Positive Emotions* (positive) and the association between *n* Affiliation and use of *W*ords >6 letters (negative), and *Present Tense* and *Positive Emotions* (positive). In contrast to these motive-specific associations, all three motive measures were consistently and negatively associated with the use of *tentative* words in both samples.

Some correlations between motive and LIWC scores were unique to each sample. In the German sample, *n* Power was negatively related to *Articles, Cognitive Processes*, and references to *Humans, Music*, or *Eating*, while it was positively related to *Negative Emotion* (which includes the *Anger* subscale), visual perception (*See*), the use of exclamation marks, and the use of uncommon words, that is, words not covered by the LIWC dictionary. There were no sample-specific correlations for *n* Power in the US sample. In the US sample, *n* Achievement was associated with fewer references to the self in the form of 1st person pronouns (*I, We, Self*), and with more *Metaphysical Issues*, particularly references to *Death*. In the German sample, *n* Achievement was associated with more *Anxiety*, visual perception *(See), School*, and *TV* and less *Insight, Humans*, and *Question Marks*. Finally, in the US sample, *n* Affiliation was associated positively with *Optimism* and *Sexuality* and negatively with *Anger* and the use of interpunctuation. In the German sample, *n* Affiliation was positively associated with the use of *Future Tense*. In the US sample, associations between PSE motive scores and LIWC categories by and large did not differ much depending on whether PSE and LIWC scores were calculated for six or eight pictures.

As expected, across both samples activity inhibition was highly correlated with *Negation*. But it was also associated positively with *Pronouns, Discrepancies, Social* processes, and general interpunctuation and negatively with *W*ords >6 Letters, Articles, Inclusion, and *Home*.

The correlations reported in Table 2 indicate that certain LIWC categories are associated with PSE motive scores in a reliable fashion, but that the size of these associations rarely exceeds the |0.35| threshold. I therefore tested whether the prediction of PSE motive scores can be improved with weighted linear combinations of LIWC scores. To do this, I regressed each 6-picture PSE motive score in each sample simultaneously only onto those LIWC categories that (1) were substantially associated with the motive score in *both samples* and that (2) accounted for additional unique portions of variance in the motive scores beyond the variance overlap they shared with other predictors. For *n* Power, *Anger* and *Tentative* fulfilled these criteria (see Table 3); for *n* Achievement, *Negations, Optimism, Tentative, Family*, and *Achievement* all contributed robustly to the prediction (see Table 4); and for *n* Affiliation, *Positive Feelings, Tentative, Social*, and *Friends* predicted motive scores (see Table 5). For all three motives, the amount of variance accounted for by the linear combinations of LIWC scores was substantially higher than for most of their zero-order correlations, ranging from *R* = 0.35 (for *n* Power in the US sample) to *R* = 0.54 (for *n* Achievement in the German sample).

**Table 3:** **Simultaneous regression of *n* Power scores (residualized for word count) on LIWC 2001 categories that are significant zero-order predictors in both samples and are non-redundant (Study 1)**.

**German sample**				**US sample**						
				**6-picture PSE**			**8-picture PSE**			
	***B***	***SE***	***p***	***B***	***SE***	***p***	***B***	***SE***	***p***	**Avg. *B***
Constant	0.540	0.264	0.04	0.276	0.284	0.33	0.082	0.301	0.78	0.408
Anger^a^	0.672	0.393	0.09	0.958	0.399	0.02	1.126	0.453	0.01	0.815
Tentat^a^	−1.060	0.262	0.0001	−0.707	0.245	0.005	−0.531	0.260	0.04	−0.883
*R*^2^	0.182			0.126			0.099			
*F*	10.79			7.91			6.03			
*df*	2, 97			2, 110			2, 110			
*P*	0.00006			0.0006			0.003			

a Log-transformed [log (1 + variable)]. Avg. B, B weight for 6-picture PSE regressions, averaged across German and US sample.

**Table 4:** **Simultaneous regression of *n* Achievement scores (residualized for word count) on LIWC 2001 categories that are significant zero-order predictors in both samples and are non-redundant (Study 1)**.

	**German sample**			**US sample**						
				**6-picture PSE**			**8-picture PSE**			
	***B***	***SE***	***p***	***B***	***SE***	***p***	***B***	***SE***	***p***	**Avg. *B***
Constant	0.090	0.481	0.85	−0.456	0.450	0.31	−0.361	0.532	0.50	−0.183
Negate	−0.419	0.138	0.003	−0.194	0.137	0.16	−0.199	0.152	0.20	−0.306
Optim	0.495	0.185	0.009	0.364	0.201	0.07	0.400	0.218	0.07	0.429
Tentat^a^	−0.340	0.257	0.19	−0.457	0.241	0.06	−0.540	0.247	0.03	−0.399
Family^a^	−0.591	0.213	0.007	−0.244	0.194	0.21	−0.347	0.204	0.09	−0.418
Achieve^a^	0.607	0.330	0.07	1.130	0.363	0.002	1.016	0.427	0.02	0.868
*R*^2^	0.287			0.221			0.234			
*F*	7.56			6.07			6.54			
*Df*	5, 94			5, 107			5, 107			
*P*	0.000005			0.00006			0.00002			

a Log-transformed [log (1 + variable)]. Avg. B, B weight for 6-picture PSE regressions, averaged across German and US sample.

**Table 5:** **Simultaneous regression of *n* Affiliation scores (residualized for word count) on LIWC 2001 categories that are significant zero-order predictors in both samples and are non-redundant (Study 1)**.

	**German sample**			**US sample**						
				**6-picture PSE**			**8-picture PSE**			
	***B***	***SE***	***P***	***B***	***SE***	***p***	***B***	***SE***	***p***	**Avg. *B***
Constant	−2.134	0.526	0.0001	−1.010	0.518	0.05	−1.177	0.570	0.04	−1.572
Posfeel^a^	1.076	0.369	0.004	0.933	0.326	0.005	0.881	0.368	0.02	1.004
Tentat^a^	−0.467	0.246	0.06	−0.627	0.225	0.006	−0.620	0.240	0.01	−0.547
Social	0.116	0.037	0.002	0.044	0.034	0.20	0.055	0.036	0.13	0.080
Friends^a^	1.178	0.455	0.01	1.721	0.403	0.00004	1.249	0.380	0.001	1.449
*R*^2^	0.281			0.275			0.244			
*F*	9.28			10.23			8.70			
*Df*	4, 95			4, 108			4, 108			
*P*	0.000002			0.0000005			0.000004			

a Log-transformed [log (1 + variable)]. Avg. B, B weight for 6-picture PSE regressions, averaged across German and US sample.

To estimate PSE motive scores from LIWC categories for further analyses, I averaged the *B* weights obtained for the 6-picture PSEs in both samples for each motive (last column in Tables 3–5) and used these average *B* weights to calculate predicted motive scores based on participants' LIWC scores. Averaged-*B* values have the advantage of being more robust and generalizable across samples than sample-specific *B* values, which provide an optimal fit for the sample they were derived from, but not for other samples. As shown in Table 6 and suggested by the regression solutions presented in Tables 3–5, LIWC-based motive scores calculated in this manner converged with the original 6-picture PSE motive scores for all three motive domains and in both samples. Moreover, in the US sample, motive scores predicted from the averaged-*B* 6-picture regression equations, but applied to LIWC scores from the 8-picture PSEs also showed substantial convergence with original 8-picture PSE motive scores (*r*s ranging from 0.30 through 0.49), which suggests that the averaged *B* weights from the 6-picture version provide comparatively robust and reliable predictions for the 8-picture PSE, too.

**Table 6:** **Multi-trait, multi-method matrix of motives coded with Winter's (1994) running-text system (residualized for word count), motive score estimations based on LIWC 2001 categories, and self-reported motives assessed with the PRF**.

	**US**	**German**	**1**	**2**	**3**	**4**	**5**	**6**	**7**	**8**	**9**	**10**	**11**	**12**	**13**	**14**	**15**	**16**	**17**	**18**	**19**
	**M (*SD*)**	**M (*SD*)**																			
1. Gender	0. 54 (0.50)	0. 49 (0.50)	−	−0.04	−	−0.12	−	0. 32^***^	−	0. 11	−	0. 13	−	0. 08	−	0. 47^***^	−	−0.24^*^	−0.13	0. 15	0. 09
2. Winter *n* Pow (6)	0. 00 (1.00)	0. 00 (1.00)	−0.08	−	−	0. 14	−	−0.18	−	−0.04	−	0. 42^***^	−	0. 17	−	0. 07	−	−0.08	0. 15	0. 03	−0.27^**^
3. Winter *n* Pow (8)	0. 00 (1.00)	−	−0.09	0. 91^***^	−	−	−	−	−	−	−	−	−	−	−	−	−	−	−	−	−
4. Winter *n* Ach (6)	0. 00 (1.00)	0. 00 (1.00)	−0.13	0. 14	0. 12	−	−	−0.02	−	−0.36^***^	−	0. 24^*^	−	0. 52^***^	−	0. 01	−	−0.03	0. 07	−0.23^*^	0. 01
5. Winter *n* Ach (8)	0. 00 (1.00)	−	−0.15	0. 18	0. 16	0. 93^***^	−	−	−	−	−	−	−	−	−	−	−	−	−	−	−
6. Winter *n* Aff (6)	0. 00 (1.00)	0. 00 (1.00)	0. 18	0. 08	0. 09	0. 15	0. 15	−	−	−0.03	−	0. 10	−	0. 14	−	0. 52^***^	−	−0.01	−0.14	0. 13	0. 10
7. Winter *n* Aff (8)	0. 00 (1.00)	−	0. 19^*^	0. 12	0. 08	0. 18	0. 14	0. 92^***^	−	−	−	−	−	−	−	−	−	−	−	−	−
8. Activity inhibition (6)	0. 00 (1.00)	0. 00 (1.00)	0. 13	0. 08	0. 06	−0.15	−0.20^*^	0. 01	0. 02	−	−	−0.26^**^	−	−0.53^***^	−	0. 05	−	0. 03	−0.15	0. 25^*^	−0.06
9. Activity inhibition (8)	0. 00 (1.00)	−	0. 16	0. 09	0. 09	−0.19^*^	−0.23^*^	−0.05	−0.03	0. 92^***^	−	−	−	−	−	−	−	−	−	−	−
10. LIWC *n* Pow (6)	−0.07 (0.39)	0. 04 (0.39)	−0.01	0. 35^***^	0. 31^***^	0. 11	0. 15	0. 09	0. 13	0. 02	0. 14	−	−	0. 49^***^	−	0. 29^***^	−	−0.04	−0.01	0. 07	0. 01
11. LIWC *n* Pow (8)	−0.08 (0.37)	−	0. 02	0. 33^***^	0. 30^***^	0. 13	0. 17	0. 11	0. 14	0. 02	0. 12	0. 95^***^	−	−	−	−	−	−	−	−	−
12. LIWC *n* Ach (6)	−0.07 (0.49)	0. 20 (0.49)	0. 08	0. 04	0. 05	0. 45^***^	0. 48^***^	0. 27^***^	0. 28^***^	−0.28^***^	−0.31^***^	0. 30^***^	0. 33^***^	−	−	0. 06	−	−0.06	−0.01	−0.09	0. 12
13. LIWC *n* Ach (8)	0. 04 (0.48)	−	0. 05	0. 04	0. 03	0. 40^***^	0. 48^***^	0. 25^**^	0. 26^***^	−0.26^***^	−0.31^***^	0. 30^***^	0. 35^***^	0. 93^***^	−	−	−	−	−	−	−
14. LIWC *n* Aff (6)	−0.02 (0.54)	0. 08 (0.50)	0. 25^*^	0. 18	0. 17	0. 05	0. 07	0. 51^***^	0. 51^***^	0. 05	−0.00	0. 23^*^	0. 25^**^	0. 32^***^	0. 31^***^	−	−	−0.05	−0.01	0. 18	0. 04
15. LIWC *n* Aff (8)	0. 17 (0.56)	−	0. 29^***^	0. 13	0. 11	0. 03	0. 01	0. 41^***^	0. 49^***^	0. 06	0. 06	0. 31^***^	0. 35^***^	0. 29^***^	0. 28^***^	0. 87^***^	−	−	−	−	−
16. PRF Dominance	9. 64 (3.50)	9. 33 (3.86)	−0.23^*^	0. 07	0. 06	0. 07	−0.02	−0.05	0. 02	0. 16	0. 10	0. 01	−0.01	−0.04	−0.01	0. 01	0. 07	0. 76/.81	0. 33^***^	0. 31^***^	0. 29^***^
17. PRF Aggression	7. 99 (3.41)	6. 21 (3.02)	−0.33^***^	0. 18	0. 17	0. 01	0. 01	−0.01	0. 00	−0.08	−0.08	0. 03	0. 04	−0.10	−0.02	−0.06	−0.11	0. 21^*^	0. 72/0.69	−0.17	−0.16
18. PRF Achievement	10. 71 (3.14)	10. 84 (2.96)	0. 13	0. 06	−0.03	0. 19^*^	0. 14	0. 14	0. 15	0. 10	0. 04	0. 03	0. 01	0. 02	−0.03	0. 05	0. 03	0. 26^**^	−0.10	0. 69/0.70	0. 26^**^
19. PRF Affiliation	11. 50 (3.13)	11. 70 (3.43)	0. 11	−0.09	−0.08	0. 12	0. 04	0. 07	0. 09	0. 00	−0.02	0. 05	0. 09	0. 13	0. 12	0. 03	0. 03	0. 21^*^	−0.08	0. 04	0. 74/0.80

Correlations for German sample above diagonal; correlations for US sample below diagonal (Study 1).

Gender: 0, male, 1, female. (6), 6-picture PSE. (8). 8-picture PSE. For US sample, pairwise n is 105 for gender × PSE, 103 for gender × PRF, 106 for PSE × PRF due to missing data for gender and PRF measures. Cronbach's alpha coefficients for PRF scales are presented on diagonal (US sample/German sample).

* *p* < 0.05,

** *p* < 0.01,

*** *p* < 0.005.

While the strategy of using only LIWC categories whose association with PSE motive scores could be obtained in both samples increases the generalizability of prediction formulas, it also incurs a loss in explained variance by ignoring LIWC correlates of motives scores that are specific to each sample and linguistic differences between English and German. I therefore also followed a second strategy for deriving regression estimations of PSE motive scores by finding linear solutions within each sample, and separately for 6- and 8-picture PSEs in the US sample, that fulfilled the following criteria: (1) LIWC category scores and PSE motive scores had significant zero-order correlations (Table 2) and (2) each LIWC category remained a significant (*p* ≤ 0.10) unique predictor when simultaneously regressed onto the content-coded PSE motive score. The resulting sample-specific prediction formulas based on the *B* weights of the final regression solutions are given in the Appendix. Not surprisingly, the number of sample-specific LIWC predictors of PSE motive scores was larger in each case than the number of predictors given for the regressions following the first strategy (cf. Tables 3–5) and therefore also converged more closely with PSE motive scores: *R*s ranged from 0.41 (*n* Power for the 8-picture PSE in the US sample) to 0.63 (*n* Affiliation for the 6-picture PSE in the US sample).

#### Discriminant validity

Like the original PSE motive scores, which did not show any substantial, replicable patterns of association with the PRF self-report measures of motivational needs, average-*B* LIWC motive scores did not significantly correlate with the PRF scales in either sample (see Table 6). When correlations between sample-specific LIWC motive scores and PRF scale scores were examined (in the US sample again both for the 6- and the 8-picture PSE), a similar picture emerged: of 36 correlations, only the one between the PRF affiliation scale and the 6-picture LIWC estimate of *n* Achievement became significant (*r* = 0.22, *p* < 0.05), and correlations between the LIWC motive measures and their conceptually corresponding PRF measures ranged from −0.12 to 0.15 (*Md* = 0.07).

LIWC scores of one motive should also be sufficiently independent of LIWC scores of a different motive, just as content-coded PSE motive scores do not show significant overlap with each other in either sample. As Table 6 shows, however, LIWC-based motive scores had substantial overlap with each other in both samples, with correlations ranging from 0.06 for *n* Achievement and *n* Affiliation scores in the German sample to 0.49 for *n* Power and *n* Achievement scores in the same sample (*Md* = 0.30). When associations between motive scores were recalculated for sample-specific LIWC estimates (6-picture PSEs), *r*s ranged from −0.24 for *n* Power and *n* Affiliation in the German sample to 0.33 for *n* Affiliation and *n* Achievement in the US sample (*Md* = 0.13). Thus, in comparison with averaged-*B* LIWC scores, sample-specific regression estimates of motives produced LIWC-based scores with better discriminant validity, because these were derived from a greater number of distinct categories.

#### Criterion validity

***Gender differences in n Affiliation.*** Table 6 shows that, like in previous studies (see Duncan and Peterson, 2010), content-coded *n* Affiliation scores were associated with gender in both samples: Women had higher scores than men. No other gender differences could be observed for content-coded motive scores in either sample. Averaged-*B* LIWC-based *n* Affiliation scores, but not *n* Achievement or *n* Power scores, also revealed this difference for both samples, and in the US sample for both 6- and 8-picture *n* Affiliation scores. For sample-specific LIWC scores, gender differences also emerged, with effect sizes (*r*) of 0.34 (6-picture PSE) and 0.38 (8-picture PSE) for the US sample and 0.43 for the German sample (all *p*s < 0.05). No other sample-specific LIWC motive scores correlated significantly with gender, *r*s ≤ |0.11|, *p*s > 0.20.

***n Agency, agentic goal progress, and emotional well-being (German sample).*** Following procedures validated in earlier studies (Brunstein et al., 1998), content-coded *n* Power and *n* Achievement scores were averaged into an overall *n* Agency variable. Similarly, rated progress on personal goals related to power and achievement were averaged into an overall agentic-goal-progress variable. When participants' self-reported emotional well-being was regressed on both variables and their interaction term, the interaction was significant (see Table 7). As in previous studies, variations in agentic goal progress were positively associated with emotional well-being only if participants were high in *n* Agency, but not if they were low in this need (see Brunstein et al., 1998). When average-*B* LIWC *n* Achievement and *n* Power scores were averaged and used as an *n* Agency measure in this regression analysis, the same significant interaction and result pattern emerged, although with a slightly smaller effect size (see Table 7). This was also true when these procedures were repeated using the sample-specific LIWC estimates of *n* Power and *n* Achievement. The overall amount of variance explained was slightly higher with the sample-specific LIWC estimates than with the LIWC estimates based on Bs from both samples.

**Table 7:** **Comparison of criterion validity of PSE scores based on Winter's (1994) coding system and scores derived from weighted LIWC dictionary scores in the German sample**.

	**Winter (1994)**			**LIWC (averaged Bs)**			**LIWC (sample-specific Bs)**		
	***B***	***SE***	***p***	***B***	***SE***	***p***	***B***	***SE***	***P***
Constant	16.613	2.712	0.000001	18.034	2.872	0.000001	16.782	2.808	0.000001
Agency	−10.538	3.745	0.006	−14.545	7.479	0.05	−13.697	6.481	0.04
Agentic goal progress	0.471	0.193	0.02	0.375	0.199	0.06	0.460	0.200	0.02
Interaction	0.695	0.256	0.008	1.007	0.514	0.05	0.847	0.431	0.05
*R*^2^	0.119			0.083			0.092		
*F*	4.34			2.89			3.26		
*Df*	3, 96			3, 96			3, 96		
*P*	0.007			0.04			0.03		
Agency	*r*_Progress_ × hedonic tone			*r*_Progress_ × hedonic tone			*r*_Progress_ × hedonic tone		
≤ Md (n = 50)	−0.02		0.89	0.11	0.45		0.13^a^		0.39
> Md (n = 50)	0.53		0.0001	0.36	0.01		0.27^a^		0.05

Agency represents averaged n Power and n Achievement scores, agentic goal progress represents averaged achievement and power goal progress scores; emotional well-being was the dependent variable (Study 1).

a To better capture the significant interaction, the sample was split slightly above the median. Therefore, n = 54 high-agency and n = 46 low-agency participants.

### Summary

Results from Study 1 show that motive scores derived through established content-coding procedures can be estimated through weighted linear combinations of LIWC 2001 categories, with overall convergent validity increasing from *n* Power to *n* Achievement and *n* Affiliation. While convergent validity was based in part on predicted marker-word categories for all three motives, these analyses also revealed unique contributions of other word categories to the prediction of motive scores. Notably, in some cases higher levels of content-coded motive scores were associated with *fewer* words represented in a category, such as fewer tentative words in the case of all three motives and fewer words related to family in the case of *n* Achievement. This finding suggests that a motivational need may not only be associated with a *preponderance* of certain story themes and the words that reflect them, but also with a *lack* of other themes and associated words. Although the absence of themes was interpreted as diagnostically revealing by previous researchers (e.g., McClelland et al., 1953), this issue has not received much attention in research since then. The present findings suggest that it should.

LIWC-derived motive scores had similar discriminant validity as content-coded motive scores vis-à-vis self-attributed motivational needs assessed with the PRF. However, they had considerably less discriminant validity relative to each other when regression solutions representative of both samples were used. Inter-motive discriminant validity improved somewhat when sample-specific LIWC estimates were used; however, as discussed in the introduction, such solutions may in turn incur a loss in generalizability. Finally, LIWC motive scores and content-coded motive scores had similar validity for two criteria: the gender difference typically seen for *n* Affiliation scores and, in the agentic domain, an interactive effect with goal progress on emotional well-being. Thus, Study 1 provides substantial evidence, most of it replicable across two samples and languages, that LIWC-derived motive scores converge with content-coded scores, predict the same criteria, and do not overlap with measures of self-attributed motivational needs.

## Study 2: the causal validity of LIWC-derived motive scores

McClelland (1958) originally proposed that a motive measure is valid to the extent that it is sensitive to variations in a motivational need. Similarly, Borsboom et al. (2004) recently argued that a measure of an attribute is valid if, and only if, it picks up causal manipulations of the attribute itself. According to these authors, convergent, discriminant, and criterion validity are not sufficient for establishing a measure's validity if such causal effects cannot be demonstrated. So how do LIWC-derived motive score estimates fare in terms of causal validity? I addressed this question by reanalyzing PSEs originally collected in a study on the effects of affiliation and power motivation arousal on hormonal changes (Schultheiss et al., 2004). In this study, participants either watched portions of an affiliation-related movie, or of a power-related movie, or of a neutral control movie, and wrote PSE stories before and after the movie as a manipulation check. Consistent with the results originally reported by Schultheiss et al. (2004) for content-coded motive scores, I expected the affiliation movie to increase LIWC-based estimates of *n* Affiliation and decrease *n* Power, the power movie to increase LIWC-based estimates of *n* Power and decrease *n* Affiliation, and the neutral movie to leave LIWC-based motive scores unchanged.

### Method

I reanalyzed data from those 30 participants (17 women; average age: 20 years) of the Schultheiss et al. (2004) study who had been administered the post-arousal PSE immediately after arousal induction. As reported by Schultheiss et al. (2004), arousal had no detectable effect in another 30 participants who had received the post-arousal PSE 30 min after the movie.

#### Design

The study had a Condition (affiliation arousal, power arousal, no arousal) × Time (PSE pre-arousal, PSE post-arousal) × Motive (*n* Affiliation, *n* Power) design. The first factor was varied between-subjects and 10 participants were randomly assigned to each of the three conditions. Affiliation arousal consisted of watching a 30-min excerpt from the romantic movie *Bridges of Madison County*, power was aroused by the presentation of 30 min from *The Godfather II*, and the neutral control condition consisted of 30 min from a documentary about the Amazon. The other design factors were within-subjects factors. In addition, PSE set sequence was balanced within each condition group, with five participants working on PSE set A (consisting of the pictures *Ship Captain, Trapeze Artists*, and *Couple Sitting Opposite a Woman*) before arousal and PSE set B (*Women in Laboratory, Nightclub Scene*, and *Girlfriends in Café*) after arousal and five participants working on the reverse sequence (first B, then A).

#### Dependent variables

The dependent variables in the original study were *n* Affiliation and *n* Power measured with Winter's (1994) running text system. Because all stories were hand-written, they were transcribed, corrected for typographical errors, and then run through the LIWC software. LIWC 2001 category scores were derived for pre- and post-arousal PSEs, and categories contributing to LIWC-based motive scores were transformed as in Study 1 to ensure comparable applicability of the regression weights for estimating scores. Because estimates were expected to be less robust for the 3-picture PSEs used in this study as compared with the 6-picture PSEs analyzed in the US and German samples, I calculated (a) US-sample-specific LIWC estimates based on the 8-picture solutions given in Tables 3 and 5 and (b) averaged-*B* estimates of *n* Affiliation and *n* Power based on the regression *B* weights given in Tables 3 and 5. The resulting scores were converted to z scores and had normal distributions in all cases.

### Results and discussion

**Figure 1:**
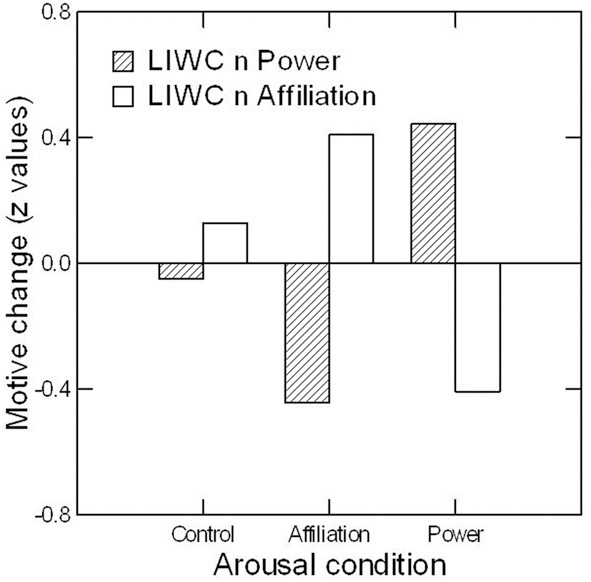
**Effect of arousal condition on changes in LIWC-estimated *n* Power and *n* Affiliation (postmovie minus premovie *z* scores) assessed immediately after motive arousal in Study 2 (*n* = 10 in each condition)**.

With PSE sequence controlled for in all analyses, there was a significant Motive × Time × Condition effect for *n* Power and *n* Affiliation based on the US sample-specific LIWC estimates (average-*B*-estimate results in parentheses), *F*_(2, 26)_ = 3.87, *p* < 0.05 [*F*_(2, 26)_ = 2.37, *p* = 0.11]. When analyses were restricted to a direct comparison of power and affiliation arousal, excluding participants in the neutral-movie control condition, the Motive × Time × Condition effect was even stronger, *F*_(1, 17)_ = 7.98, *p* = 0.01 [*F*_(1, 17)_ = 4.83, *p* < 0.05], similar to the results reported by Schultheiss et al. (2004) for motive scores based on content coding. As Figure 1 illustrates for US-sample-specific LIWC estimates of motive scores, the three-way interaction was due to participants showing an increase in *n* Affiliation and a decrease in *n* Power in response to affiliation arousal, but a decrease in *n* Affiliation and an increase in *n* Power in response to power arousal. There was almost no change in either score in the neutral control condition. The overall pattern of findings closely resembles the one for the content-coded scores originally reported and depicted by Schultheiss et al. (2004). When analyzed separately for *n* Affiliation and *n* Power, the difference in LIWC-estimated *n* Affiliation changes to affiliation vs. power arousal was significant, as indicated by a Time × Condition interaction, *F*_(1, 17)_ = 5.86, *p* < 0.05, [*F*_(1, 17)_ = 5.56, *p* < 0.05]. The difference in LIWC-estimated *n* Power failed to become significant, *F*_(1, 17)_ = 2.84, *p* = 0.11 [*F*_(1, 17)_ = 0.48, *p* = 0.50].

How much did LIWC-estimated motive score changes covary with content-coded *n* Power and *n* Affiliation? To address this question, I computed, for all 30 individuals, bipartial correlation coefficients (see Cohen and Cohen, 1983) for post-arousal scores, partialling the respective pre-arousal motive scores as well as PSE sequence and post-motive word count from each measure, as these variables were significantly correlated with post-arousal scores in most cases. In the case on *n* Affiliation, the bipartial *r* between LIWC- and coding-derived scores was 0.45, *p* < 0.05 (0.43, *p* < 0.05). For *n* Power, bipartial *r* = 0.41, *p* < 0.05 (0.28, *p* = 0.15).

To summarize, LIWC-estimated motive scores were sensitive to experimental arousal of *n* Affiliation and *n* Power, with the effect being stronger for *n* Affiliation than for *n* Power. Increases in LIWC-estimated motive scores were reliably related to increases in content-coded motive scores. Both sets of findings emerged equally for sample-specific and averaged-*B* LIWC estimates of *n* Affiliation and for sample-specific LIWC estimates of *n* Power. These findings suggest that LIWC estimates of *n* Affiliation and, to a lesser extent, *n* Power reflect causal effects of motive arousal.

## General discussion

This research aimed at testing the marker-word hypothesis, which states that there are types of words whose frequency is diagnostic of individual differences in an implicit motive. Using the LIWC 2001 dictionary and software to capture a diverse range of linguistic features, I analyzed PSEs from three samples to derive and validate linear combinations of word categories that sensitively and robustly converge with content-coded measures of *n* Power, *n* Achievement, and *n* Affiliation, three frequently measured motive dispositions.

In Study 1, I subjected PSEs from German and US American samples to LIWC analysis and identified correlations between LIWC categories and motive scores derived with Winter's (1994) integrated content-coding system that emerged consistently in both samples. Although the identified individual correlation coefficients were in the small-to-medium range, linear combinations of LIWC categories identified through multiple regression analysis and based on *B* weights averaged across both samples showed better convergence with content-coded scores, with effect sizes ranging from medium to large. Study 1 also demonstrated that LIWC-based motive scores have good discriminant validity when compared to a standard measure of explicit motives (PRF). However, compared to content-coded motive measures, LIWC-based scores have considerably lower within-method discriminant validity. This problem could be alleviated to some extent when sample-specific regression solutions, as opposed to solutions based on *B* averaged across samples, were used. Study 1 also provided some evidence for the criterion validity of LIWC-based motive scores: Across both samples, LIWC-based *n* Affiliation scores showed the same specific gender effect that was also observed for content-coded *n* Affiliation in this and in previous research. Moreover, in the German sample, high LIWC-based agency scores (i.e., the sum of *n* Power and *n* Achievement scores), in conjunction with high progress on agentic goals, was associated with enhanced emotional well-being, paralleling similar effects for agency motivation based on content-coding found in this and previous research.

In Study 2, I tested the causal validity of LIWC-based motive scores by examining their sensitivity to experimentally induced arousal of the motivational needs for power and affiliation. The presentation of movies with affiliative or power-related content, relative to a control movie with motivationally neutral content, elicited corresponding changes in LIWC-derived estimates of *n* Affiliation and *n* Power from before to after the movie. Moreover, changes in LIWC-based motive scores correlated with changes in content-coded motive scores.

Taken together, these findings provide support for the marker-word hypothesis by demonstrating that motive scores based on content coding can be approximated with satisfactory convergent validity and replicability through weighted linear combinations of LIWC's word categories, that LIWC-based scores have acceptable discriminant validity, and that they show evidence of criterion and causal validity.

In general, convergent validity between LIWC-based scores and content-coding scores were higher in the present research than in Pennebaker and King's (1999) earlier study. The previously discussed differences in analytic approach notwithstanding (factor scores in their study vs. linear weighted category combinations in the present research), Pennebaker and King (1999) obtained the least convergence between LIWC and content-coding scores for the domain of *n* Power, a result that parallels the present findings. However, whereas these authors report essentially no reliable variance overlap between LIWC factor scores and content-coded *n* Power, a reliable overlap was found here when *n* Power was assessed as a disposition (Study 1) or as a changing state (Study 2). Yet, in Study 1 convergence for *n* Achievement and *n* Affiliation was even stronger and based on more word categories. This suggests that the LIWC 2001 dictionary may be better suited for capturing words specifically related to these motives than to *n* Power. Perhaps the LIWC dictionary, whose *Anger* scale was the only category specifically associated with *n* Power across US and German samples, needs to be complemented in future research by additional word categories that capture more subtle manifestations of *n* Power, such as words related to arguing, persuading and convincing, or words associated with eliciting strong emotions, such as surprise, shock, awe, etc (see the coding categories sketched out in the introduction).

How robustly do the linear combinations of LIWC categories derived in the present research converge with content-coded motive scores? How much do they depend on the specific PSE used in Study 1 to derive them and could they also be used with other types of text material to assess a person's motives? The present research, although limited to three samples, suggests that the linear combinations are surprisingly robust. They yielded scores that not only correlated with content-coded motive scores in a similar way in two samples from different cultures and with different languages. Study 2 also provided evidence that the LIWC score formulas derived with the PSE used in Study 1 can be applied to a shorter PSE with slightly different pictures without losing their validity. Moreover, a recent study on *n* Achievement and memory by Bender et al. (2012) suggests that the LIWC 2001 categories identified in the present search also emerge for text material elicited by means other than the PSE. These researchers found the content-coded *n* Achievement assessed with the PSE correlated positively with the *Achievement* and *Work* categories and negatively with the *Social* category derived from an analysis of daily memories using LIWC 2007 (Pennebaker et al., 2007). These findings converge with the observation of Study 1 that content-coded *n* Achievement is positively associated with *Achievement* and *Occupation* (incorporated into *Work* in LIWC 2007) and negatively with *Family*, a subcategory of *Social* words.

While more research is needed to test the validity of the marker-word approach to motive assessment in a sufficiently stringent manner, these observations provide initial evidence for robust generalizability and validity of the LIWC-based motive score estimates derived in the present research. They suggest that if content-coding is not feasible, LIWC analysis of texts using the regression formulas provided in Tables 3–5 and in the Appendix may provide rough, but valid proxy measures of implicit motives.

How far can the marker-word approach be taken? Is it conceivable that it could replace more traditional content-coding methods or is too much information lost when mere word frequencies instead of complex semantic information are used for motive assessment? Clearly, the word-count approach misses out on subtle contexts and semantic contingencies that render a sentence codeable or uncodeable with traditional content-coding methods. The observation that convergence coefficients are in the medium effect size range suggests that the marker-word approach captures only some aspects but by no means all of what content-coding methods identify as motive imagery. However, it should also be noted that the size of these coefficients is not substantially lower than the convergence coefficients of 0.45–0.72 Winter (1991, Table 2) reports for scores obtained with his integrated running-text coding system and scores obtained from the same text materials using previous coding systems. This finding suggests that even among traditional content-coding methods, convergence is not perfect, and a sentence or phrase that is coded for a given motive with one system may not always be coded with another, alternative coding system.

Convergence between the LIWC categories and motive scores derived with Winter's (1994) running-text system may also be attenuated by the second-sentence coding rule of this coding system. According to this rule, imagery for a particular motive that is present in two consecutive sentences may only be coded in the first sentence. Since this rule is not implemented in the LIWC software, motive-marker words would also be counted in the second sentence. The rule may therefore have restricted the degree of convergence theoretically possible between content-coding and word-count approaches. Despite these potential sources of attenuated score validity, both Winter's (1994) running-text system and previous coding systems have an established track record of predictive and criterion validity, and the marker-word approach espoused here also shows robust validity for two well-established criteria of implicit motives, that is, the gender difference in *n* Affiliation and motive × goal-progress effects on emotional well-being.

Moreover, the approach used in the present research has also helped to uncover aspects of language that are associated with content-coded motive scores and contribute to LIWC motive estimates' validity, but that have never been explicitly identified as critical by researchers devising content-coding measures. Take the LIWC *Tentative* category as a case in point. According to coding systems by Winter (1994) and others (see Smith, 1992), tentative statements such as “Perhaps he wants blackmail his boss” or “I guess they are in love” would be coded for power and affiliation, respectively, because they feature relevant imagery and are not negated. Yet the findings obtained in the present research suggest that the more tentative words (e.g., *perhaps, guess*) a PSE writer uses, the lower his or her content-coded motive scores for all three motives are going to be. In contrast, writers high in implicit motives use less tentative language. This finding suggests that there may be hidden aspects of language, perhaps particularly the *absence* of certain words, that are diagnostic of a given motive and that could be uncovered by the marker-word approach. More generally, I suggest that an analysis of the language used in PSE stories may reveal linguistic features that content-coding systems do not capture but that may provide important diagnostic information about motivational needs.

One shortcoming of the present approach is its reliance on convergence of LIWC categories with content-coded motive scores. Although this approach has served as a good starting point for parsing linguistic dimensions that are reliably associated with motive scores across samples, languages, and PSEs, the causal relationship between aroused motivation and LIWC category variations remains an issue. The LIWC regression formulas derived in the present research may correlate to some unknown extent with variance portions of content-coded motive scores that do not directly reflect a causative influence of motivation on story writing. Study 2 suggests that if this problem exists, it is not extensive, at least not for the assessment of *n* Affiliation and *n* Power, because experimental arousal elicited predictable changes in LIWC-based scores for both motives. Although the design of Study 2 was not suitable to test whether the LIWC-based *n* Achievement score is also sensitive to experimental arousal, a recent study by Shantz and Latham (2009) suggests that it might, at least some facets of it. These researchers either aroused *n* Achievement by presenting a picture of a woman finishing a marathon race or left it unaroused by presenting a control picture and had participants write imaginative stories about three novel picture cues. Stories were analyzed with LIWC specifically for the *Achievement* category. Aroused participants had higher scores than control-group participants. Thus, one of the core categories constituting the LIWC-based *n* Achievement score derived in the present research is sensitive to motivational arousal.

I suggest that a particularly fruitful direction for future work on the marker-word approach is to study the effects of experimentally aroused motivation on changes in language on the PSE, as assessed with the LIWC dictionary and perhaps with additional, custom-tailored dictionaries and sophisticated automated analysis tools, such as latent semantic analysis (see Landauer et al., 2007). An experimental-manipulation approach, which is viewed as producing critical evidence for a measure's validity (see Borsboom et al., 2004), may help to separate the wheat from the chaff and identify those linguistic markers that reliably differentiate between individuals whose motivational need is aroused from those whose need has not been aroused. Such an approach can help weed out those LIWC categories that may correlate with content-coding measures, but that are not sensitive to motivational arousal and thus not valid indicators of the motive in question. And it can help identify additional aspects of language that sensitively pick up effects of aroused motivation, but that may not correlate substantially with content-coding measures of motives. Eventually, such studies will allow comparing effect sizes between content-coding measures and linguistic measures and help to decide which approach yields the more valid motive measures in the end.

## Conclusion

The present research suggests that the marker-word hypothesis has merit. Assessment of implicit motives with a word-count approach yields scores that converge with content-coded motive measures, that predict well-documented validation criteria of implicit motive measures, and that respond sensitively to experimental arousal of motivation. The marker-word approach corroborates the idea that certain word categories should be associated with specific motive measures for a priori reasons (e.g., words related to anger, achievement, and friendship). But it also allows the a-posteriori exploration and identification of additional dimensions of language that are diagnostic of motivational dispositions. The present research thus demonstrates that the obstacles that have hampered earlier tests of the marker-word hypothesis can be overcome through the ubiquitous availability of high-speed computers, well-validated and accessible software, and the development of word-count measures that have sufficient generalizability to be useful across different languages and story-eliciting cues. I believe that this approach holds great promise for the evolution of motive assessment and should be explored broadly and vigorously in future research.

### Conflict of interest statement

The author declares that the research was conducted in the absence of any commercial or financial relationships that could be construed as a potential conflict of interest.

## Appendix

Sample-specific regression solutions for predicting PSE motive scores (Winter, 1994) with LIWC 2001 dictionary categories. *R* represents the multiple correlation and the Pearson correlation between LIWC-estimated motive scores and actual scores (*z* values). Log-transformations are denoted as superscript l, dichotomized variables are denoted as superscript d.

### Us sample

#### n Power

(1) 6 pictures: −0.654 + 0.653^*^Anger^1^ − 0.678^*^Tentative^1^ + 0.247^*^Space + 0.640^*^Money^1^

*R* = 0.44, all *p*s ≤ 0.10, *F*_(4, 108)_ = 6.52, *p* = 0.0001

(2) 8 pictures: −0.293 + 0.812^*^Anger^1^ − 0.532^*^Tentative^1^ + 1.326^*^Down^d^ + 0.763^*^Money^1^

*R* = 0.41, all *p*s ≤ 0.10, *F*_(4, 108)_ = 5.35, *p* = 0.0006

#### n Achievement

(3) 6 pictures: −0.858 − 0.631^*^Self^d^ − 0.245^*^Negate + 0.322^*^Optimism + 1.071^*^Achievement^1^ + 0.479^*^Metaphysical Issues^d^

*R* = 0.58, all *p*s ≤0.10, *F*_(5, 107)_ = 10.80, *p* = 0.0000005

(4) 8 pictures: −0.998 − 0.521^*^Self^d^ − 0.325^*^Negate + 0.357^*^Optimism + 1.059^*^Achievement^1^ + 0.569^*^Metaphysical Issues^d^

*R* = 0.57, all *p*s ≤ 0.10, *F*_(5, 107)_ = 10.31, *p* = 0.0000005

#### n Affiliation

(5) 6 pictures: −1.857 + 0.206^*^Other + 0.870^*^Positive Feelings^1^ − 0.106^*^Social + 1.557^*^Friends^1^ + 0.047^*^Present + 0.961^*^Sexuality^d^ − 0.377^*^Question Mark^d^

*R* = 0.63, all *p*s ≤ 0.05, *F*_(7, 105)_ = 10.02, *p* = 0.0000005

(6) 8 pictures: −1.407 + 0.140^*^Other − 1.075^*^Anger^1^ + 1.066^*^Friends^1^ + 1.384^*^Sexuality^d^ − 0.507^*^Question Mark^d^

*R* = 0.60, all *p*s ≤ 0.01, *F*_(5, 107)_ = 12.23, *p* = 0.0000005

### German sample

#### n Power

(7) 4.309 + 0.212^*^Negative Emotions − 0.841^*^Tentative^1^ + 0.317^*^See^d^ + 0.335^*^Exclamation Mark^d^ − 0.062^*^Dictionary

*R* = 0.54, all *p*s ≤ 0.10, *F*_(5, 94)_ = 7.76, *p* = 0.000004

#### n Achievement

(8) 0.167 − 0.326^*^Negate + 0.490^*^Optimism − 0.210^*^Insight − 0.582^*^Family^1^ + 0.808^*^Achievement^1^ − 0.425^*^Question Mark^d^

*R* = 0.59, all *p*s ≤ 0.05, *F*_(6, 93)_ = 8.07, *p* = 0.000001

#### n Affiliation

(9) −1.403 − 0.061^*^Words > 6 letters + 0.066^*^Other + 0.127^*^Positive Emotions + 0.838^*^Positive Feelings^1^ + 0.346^*^Communication + 1.384^*^Friends^1^ + 0.625^*^Future^1^

*R* = 0.61, all *p*s ≤ 0.10, *F*_(7, 92)_ = 7.59, *p* = 0.0000005
